# American Civil War plant medicines inhibit growth, biofilm formation, and quorum sensing by multidrug-resistant bacteria

**DOI:** 10.1038/s41598-019-44242-y

**Published:** 2019-05-22

**Authors:** Micah Dettweiler, James T. Lyles, Kate Nelson, Brandon Dale, Ryan M. Reddinger, Daniel V. Zurawski, Cassandra L. Quave

**Affiliations:** 1 Center for the Study of Human Health, Emory University, Atlanta, Georgia USA; 2 Department of Dermatology, Emory University School of Medicine, Atlanta, Georgia USA; 3 Wound Infections Department, Bacterial Diseases Branch, Walter Reed Army Institute of Research (WRAIR), Silver Spring, Maryland USA; 4 Emory Antibiotic Resistance Center, Emory University, Atlanta, Georgia USA; 5 Emory University Herbarium, Emory University, Atlanta, Georgia USA

**Keywords:** Antibiotics, Secondary metabolism

## Abstract

A shortage of conventional medicine during the American Civil War (1861–1865) spurred Confederate physicians to use preparations of native plants as medicines. In 1863, botanist Francis Porcher compiled a book of medicinal plants native to the southern United States, including plants used in Native American traditional medicine. In this study, we consulted Porcher’s book and collected samples from three species that were indicated for the formulation of antiseptics: *Liriodendron tulipifera*, *Aralia spinosa*, and *Quercus alba*. Extracts of these species were tested for the ability to inhibit growth in three species of multidrug-resistant pathogenic bacteria associated with wound infections: *Staphylococcus aureus*, *Klebsiella pneumoniae*, and *Acinetobacter baumannii*. Extracts were also tested for biofilm and quorum sensing inhibition against *S. aureus. Q. alba* extracts inhibited growth in all three species of bacteria (IC_50_ 64, 32, and 32 µg/mL, respectively), and inhibited biofilm formation (IC_50_ 1 µg/mL) in *S. aureus*. *L. tulipifera* extracts inhibited biofilm formation (IC_50_ 32 µg/mL) in *S. aureus*. *A. spinosa* extracts inhibited biofilm formation (IC_50_ 2 µg/mL) and quorum sensing (IC_50_ 8 µg/mL) in *S. aureus*. These results support that this selection of plants exhibited some antiseptic properties in the prevention and management of wound infections during the conflict.

## Introduction

Antibiotic resistance in pathogenic microbes poses a significant threat to human health^1^; antibiotics are critical not only in treating bacterial diseases but also in enabling surgery and other procedures with high risks of infection. Given the great genetic diversity and capacity for evolution present in bacteria, a rise in antibiotic resistance is an inevitable response to antibiotic use. For example, in 1940, even before penicillin was widely used, penicillin resistance was observed. Any single antibiotic, then, is not a permanent solution but another step in the struggle against infection.

Several factors complicate the relationship between antibiotics and bacteria. For example, the innate immune system plays a role in fighting infections with or without the use of antibiotics. Further, commensal members of the microbiome may compete with pathogenic bacteria or may themselves become pathogenic under certain circumstances. Relevant to this study, bacterial community effects such as biofilms and quorum sensing produce resistance and virulence phenotypes not necessarily observed *in vitro*^2,3^. Biofilms are extracellular mixtures of polysaccharides and proteins that can physically protect bacterial populations from antibiotics and immune responses^2,4^. Consequently, biofilms are associated with chronic infections, especially in the cases of indwelling medical devices and implants, and there is currently a lack of effective treatments for these conditions^4^. Quorum sensing is a system by which toxin production or other pathogenic activity is initiated when extracellular communication indicates achievement of a threshold population of bacteria. Inhibition of quorum sensing and biofilm formation, then, can be therapeutic but not bactericidal^3^. In the absence of new antibiotics, multidrug-resistant infections may be treatable by administering biofilm inhibitors or quorum quenchers to increase the vulnerability of bacteria to the immune system or conventional antibiotics.

The natural product compositions investigated in this study are plant extracts used during the American Civil War (1861–1865), a period of history in which infections were treated without the use of modern antibiotics and before the emergence of germ theory. The accepted definition of antiseptic was “tonic useful to prevent external or internal mortification”^5^. Union General Ulysses S. Grant once famously demanded that onions be sent to him before he would move his army. While soldiers certainly used onions in their cooking, we know now that antimicrobial agents such as ajoene and allicin found in garlic and onions have an impact on quorum sensing and biofilm to disrupt infections^6,7^. At the time, they were used to treat powder burns.

**Figure 1:**
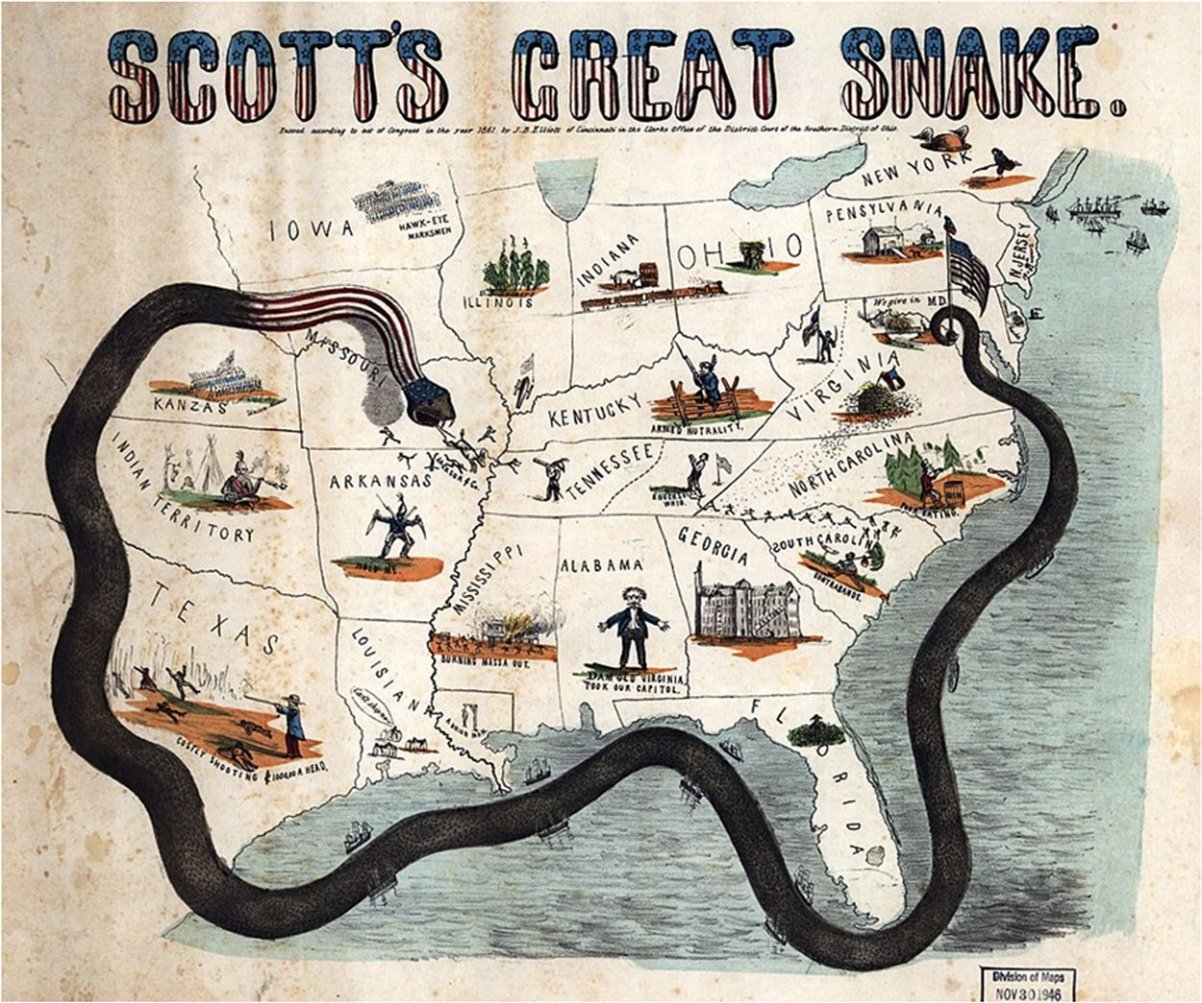
Cartoon map of the Union blockade proposed by General Winfield Scott during the American Civil War. Created in 1861 by J.B. Elliott and entered in Library of Congress, Geography and Map Division.

During the latter half of the war, a Union blockade^8^ prevented the Confederacy from importing sufficient amounts of conventional medicines such as quinine, morphine, and chloroform (Fig. 1)^9^. Francis Porcher, a botanist, was commissioned to find and catalogue plants native to the southeastern US that could be used as medicines in their place^5^. Porcher compiled a book of his findings, including 37 plant species to be used as antiseptics, treating gangrene and other infections^5^. From this research, Samuel Moore, the Confederate Surgeon General, published a field guide of native plant medicines to be used by battlefield physicians, including methods of collection, preparation, and administration^10^. Infection was a leading cause of death for soldiers in the Civil War and was often treated with amputation^11^. It may be hoped that Porcher’s work with medicinal plants saved many lives and limbs.

Natural products—compounds produced by living organisms—are used directly as medicine by an estimated 4 billion people for whom traditional medicine is a primary healthcare source^12^. Approximately 25% of modern drugs are derived from natural products used in traditional medicine^13^. Plants in particular produce a large variety of secondary metabolites to interact with their environments, and some of these serve to control local microbes by encouraging or inhibiting bacterial growth and/or function.

Many of the plant species Porcher described as antiseptic have not been tested for antibiotic activity, particularly for adjuvant activity (biofilm and/or anti-virulence properties) or activity against multidrug-resistant bacteria. The aim of this study was to examine the potential efficacy of the plants used to stave off infection during the Civil War. While the majority of drugs on the market today are synthetic, many are still derived from natural products; a review of new drugs from 1981–2014 found that only two new approved novel chemical entities (NCEs), sorafenib and ataluren, were created through *de novo* combinatorial chemistry^13^. Searching natural products for NCEs may be a more effective tactic, especially when systems of traditional medicine and historical pharmacopoeias are available to use as heuristics.

One benefit of natural product extracts as antibiotic agents over single-compound drugs is that due to the presence of dozens to thousands of compounds, they can exhibit multiple mechanisms of activity, potentially making it more difficult for resistance to develop. For example, English oak (*Quercus robur* L., Fagaceae) bark was found to exert its quorum quenching activity via two distinct mechanisms^14^.

In this study, samples of three species from Porcher’s book were selected for investigation: white oak (*Quercus alba* L., Fagaceae), devil’s walking stick (*Aralia spinosa* L., Araliaceae), and tulip tree (*Liriodendron tulipifera* L., Magnoliaceae). We hypothesized that, given the historic use of these plants as antiseptics, their extracts may inhibit growth, biofilm production, and/or quorum sensing in pathogenic bacteria that affect the skin and soft tissue structures. Multidrug-resistant bacteria were used in all experiments to examine the potential use of these plant compounds to combat emerging resistance in species commonly found in wound infections today.

## Results

### Extract yield

Extraction in MeOH yielded six crude extracts, representing *Q. alba* bark and galls, *A. spinosa* leaves, and *L. tulipifera* leaves, root inner bark, and branch bark (Table 1). Extract yield was highest (27.1% of dry mass) in extract 620 (*Q. alba* galls). Other crude extracts had yields ranging from 8–11%. Masses of partitions and fractions of crude extracts varied from <0.1% to 4% relative to dry plant matter (Supplementary Table S1). Partitions were labelled B, C, D, and E for solvents hexane, ethyl acetate, *n*-butanol, and water, respectively; non-tannin fractions were labelled F1 and tannin fractions were labelled F2. The non-tannin fraction of *L. tulipifera* leaves (616-F1) was more than 10 times as massive as the tannin fraction, suggesting that tannin content is not high in *L. tulipifera* leaves. The tannin and non-tannin fractions of *Q. alba* bark were similar in mass.

**Table 1:** Preparation of plant materials.

Botanical Name (Voucher #)	Plant Part	Drying Procedure	Grinding Procedure	Extract Number	Percent Yield
*Aralia spinosa* L. (MD-023)	leaves	drying cabinet	Wiley Mill with 2 mm mesh	618	10.92
*Liriodendron tulipifera* L. (MD-027)	leaves	drying cabinet	Wiley Mill with 2 mm mesh	616	10.71
	root inner bark	cut into 3 × 3 cm pieces, drying cabinet	Wiley Mill with 2 mm mesh	617	8.39
	branch inner bark	cut into 1 × 3 cm pieces, drying cabinet	coffee grinder	621	9.65
*Quercus alba* L. (MD-022)	bark	cut into 3 × 3 cm pieces, drying cabinet	Wiley Mill with 2 mm mesh	619	8.78
	branch galls	drying cabinet	coffee grinder	620	27.10

### Growth inhibition

All 19 crude extracts, partitions, and fractions were tested for growth inhibition of *S. aureus*, *A. baumannii*, *K. pneumoniae*, and *P. aeruginosa* (Table 2). Reported here are minimum concentrations of extract that achieved 50% inhibition (IC_50_) and 90% inhibition (MIC or IC_90_). Extracts from *L. tulipifera* and *Q. alba* were shown to be most active in inhibition of *S. aureus* growth (IC_50_ 64 μg/mL in each case). *Q. alba* extracts 619, 619-F2, and 620 displayed inhibition of *A. baumannii* (IC_50_ 64, 32, and 32 μg/mL, respectively) and *K. pneumoniae* (IC_50_ 128, 64, and 32 μg/mL).

**Table 2:** Growth inhibition of multidrug-resistant bacteria by Civil War samples.

Sample	Species	*S. aureus*		*A. baumannii*	*K. pneumoniae*	*P. aeruginosa*
	Strain	UAMS1	NRS385	EU27	EU32	AH71
616	IC_50_	>256	256	>256	>256	>256
	MIC	>256	>256	>256	>256	>256
616-F1	IC_50_	256	256	>256	>256	>256
	MIC	>256	>256	>256	>256	>256
616-F2	IC_50_	>256	>256	>256	>256	>256
	MIC	>256	>256	>256	>256	>256
617	IC_50_	128	256	>256	>256	>256
	MIC	>256	>256	>256	>256	>256
617B	IC_50_	64	128	>256	>256	>256
	MIC	256	256	>256	>256	>256
617 C	IC_50_	128	128	>256	>256	256
	MIC	>256	>256	>256	>256	>256
617D	IC_50_	>256	>256	>256	>256	>256
	MIC	>256	>256	>256	>256	>256
617E	IC_50_	>256	256	>256	>256	>256
	MIC	>256	>256	>256	>256	>256
618	IC_50_	>256	256	>256	>256	>256
	MIC	>256	>256	>256	>256	>256
618B	IC_50_	128	128	>256	>256	>256
	MIC	>256	>256	>256	>256	>256
618 C	IC_50_	>256	128	>256	>256	>256
	MIC	>256	>256	>256	>256	>256
618D	IC_50_	>256	>256	>256	>256	>256
	MIC	>256	>256	>256	>256	>256
618E	IC_50_	>256	>256	>256	>256	>256
	MIC	>256	>256	>256	>256	>256
619	IC_50_	128	256	64	128	>256
	MIC	256	256	>256	>256	>256
619-F1	IC_50_	>256	256	>256	>256	>256
	MIC	>256	>256	>256	>256	>256
619-F2	IC_50_	64	128	32	64	128
	MIC	128	128	>256	>256	>256
619 W	IC_50_	>256	—	>256	>256	>256
	MIC	>256	—	>256	>256	>256
620	IC_50_	128	>256	32	32	64
	MIC	>256	>256	>256	>256	256
620 W	IC_50_	64	—	32	>256	—
	MIC	>256	—	>256	>256	—
621	IC_50_	>256	256	>256	>256	>256
	MIC	>256	>256	>256	>256	>256
Amp	IC_50_	>256	>256	—	>256	—
	MIC	>256	>256	—	>256	—
Kan	IC_50_	2	>256	—	—	—
	MIC	4	>256	—	—	—
Van	IC_50_	4	8	—	—	—
	MIC	8	8	—	—	—
Gent	IC_50_	—	—	64	0.5	0.5
	MIC	—	—	>256	0.5	0.5
Tet	IC_50_	—	—	2	4	—
	MIC	—	—	4	8	—

IC_50_ and MIC (IC_90_) values are expressed as concentration (μg/mL), with a maximum concentration tested of 256 μg/mL. Dashes indicate that a sample was not tested.

Extracts which displayed strong activity against *S. aureus*, *A. baumannii*, *K. pneumoniae*, and *P. aeruginosa* (619, 619-F2, and 620) were tested for growth inhibition of *S. epidermidis* and additional strains of *A. baumannii* and *K. pneumoniae*. All three of these *Q. alba* extracts inhibited growth in the strains of *A. baumannii* (IC_50_ 32–256 μg/mL), but not in the additional *K. pneumoniae* strains tested (Table 3). *Q. alba* extracts 619 and 619-F2 were found to inhibit growth of *S. epidermidis* (IC_50_ 256 and 64 μg/mL, respectively).

**Table 3:** Growth inhibition of additional strains by *Q. alba* samples 619, 619-F2, and 620.

Sample		*A. baumannii*						*K. pneumoniae*			*S. epidermidis*
		EU24	AB5075	CDC0035	CDC0037	CDC0045	CDC0300	EU33	EU34	EU36	NRS101
619	IC_50_	128	256	>256	>256	>256	256	>256	>256	>256	256
	MIC	>256	>256	>256	>256	>256	>256	>256	>256	>256	>256
619-F2	IC_50_	128	64	>256	128	128	64	>256	>256	>256	64
	MIC	>256	>256	>256	>256	>256	>256	>256	>256	>256	>256
620	IC_50_	32	32	64	32	64	32	>256	>256	>256	>256
	MIC	>256	>256	>256	>256	>256	>256	>256	>256	>256	>256
Amp	IC_50_	—	—	—	—	—	—	>256	>256	>256	64
	MIC	—	—	—	—	—	—	>256	>256	>256	64
Kan	IC_50_	—	—	—	—	—	—	—	—	—	>256
	MIC	—	—	—	—	—	—	—	—	—	>256
Van	IC_50_	—	—	—	—	—	—	—	—	—	16
	MIC	—	—	—	—	—	—	—	—	—	16
Gent	IC_50_	—	—	—	—	—	—	16	2	64	—
	MIC	—	—	—	—	—	—	32	2	64	—
Tet	IC_50_	—	—	—	—	—	—	1	4	2	—
	MIC	—	—	—	—	—	—	4	4	4	—
Mem	IC_50_	1	32	>256	>256	>256	2	—	—	—	—
	MIC	2	32	>256	>256	>256	2	—	—	—	—

IC_50_ and MIC values are expressed as concentration (μg/mL), with a maximum concentration tested of 256 μg/mL. Dashes indicated that a sample was not tested.

### Biofilm inhibition

**Figure 2:**
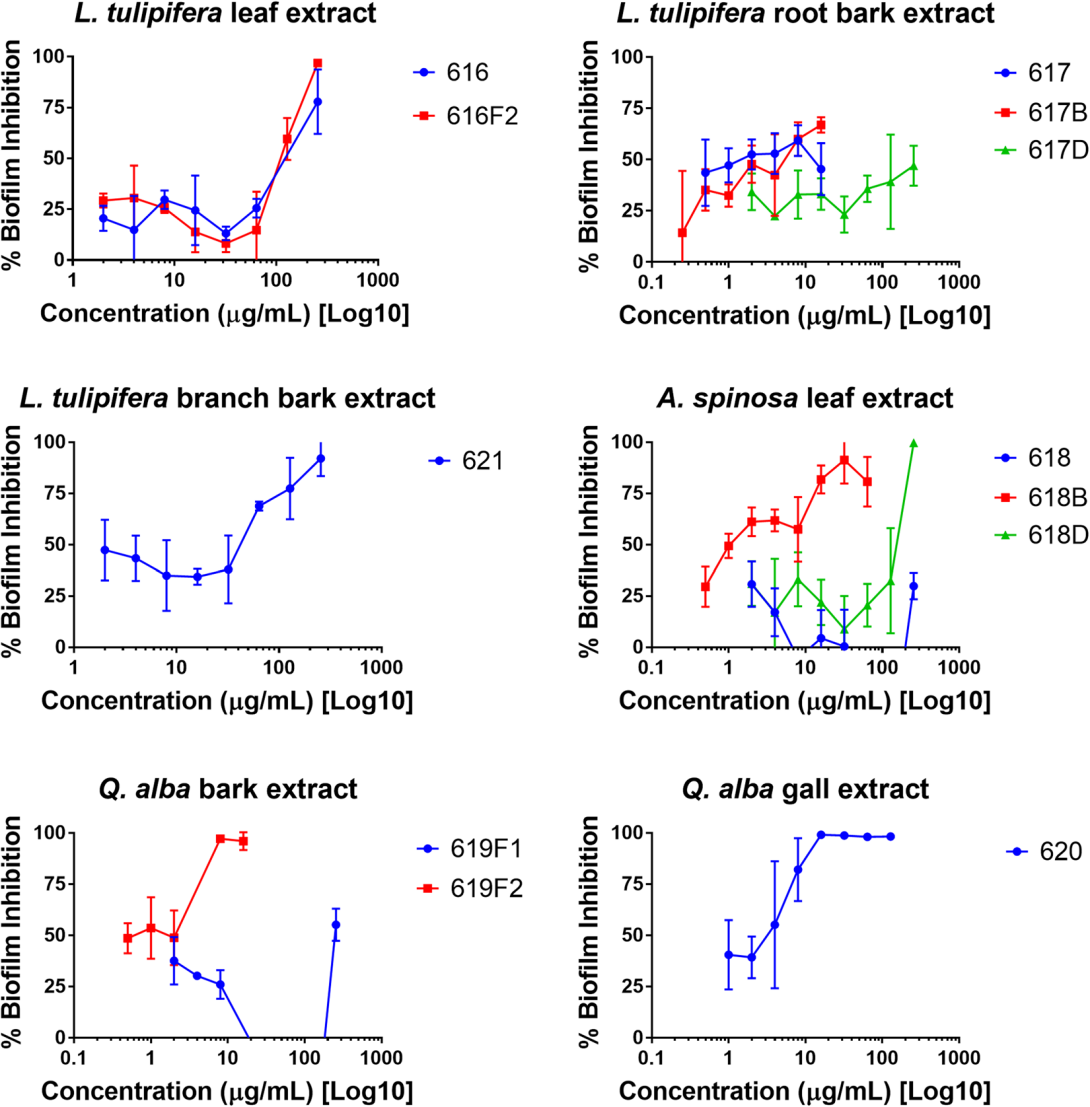
Biofilm inhibition of *S. aureus* by Civil War samples. Extracts tested at sub-IC_50_ concentration. Percent biofilm inhibition calculated as inhibition compared to vehicle control.

Extracts from all species inhibited *S. aureus* biofilm formation (IC_50_ 1–256 µg/mL). Figure 2 shows biofilm inhibition across serial dilutions of the most active extracts. *Q. alba* extract 619-F2 displayed the greatest biofilm inhibition (IC_50_ 1 µg/mL). Some extracts, such as *L. tulipifera* extract 616-F1 and *A. spinosa* extract 618B, displayed little growth inhibition activity against *S. aureus* but strongly inhibited biofilm formation (IC_50_ 32 and 2 µg/mL, respectively). Full biofilm inhibition data is reported in Supplementary Table S2.

### Quorum sensing inhibition

**Figure 3:**
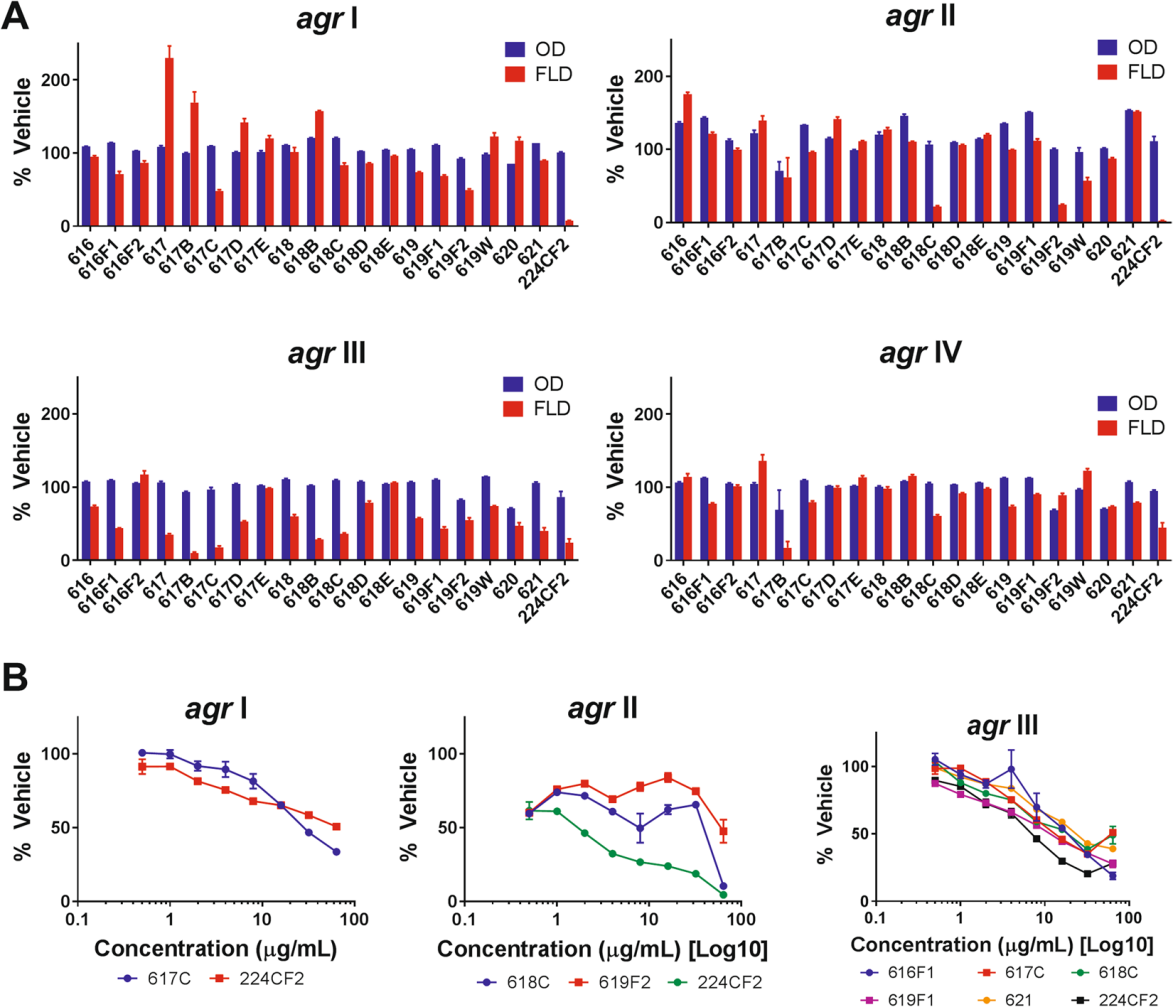
Quorum sensing inhibition of *S. aureus* by Civil War samples. (**A**) Screen of all samples at 64 µg/mL. OD represents *S. aureus* growth and FLD represents expression of the *agr* gene. (**B**) Serial dilution of active samples from 0.5 to 64 µg/mL. Only 224C-F2, the control, showed activity against *agr* IV at sub-inhibitory concentrations for growth.

Transcription of *S. aureus agr* types I, II, and III was inhibited by several Civil War extracts (Fig. 3). *L. tulipifera* extract 617 C, *A. spinosa* extract 618 C, and *Q. alba* extract 619-F1 exhibited the most activity in these assays, primarily against *agr* III (IC_50_ 16, 32, and 16 μg/mL, respectively). No extracts demonstrated inhibition of *agr* IV transcription. Full quorum sensing inhibition data is reported in Supplementary Table S3.

### Cytotoxicity

**Figure 4:**
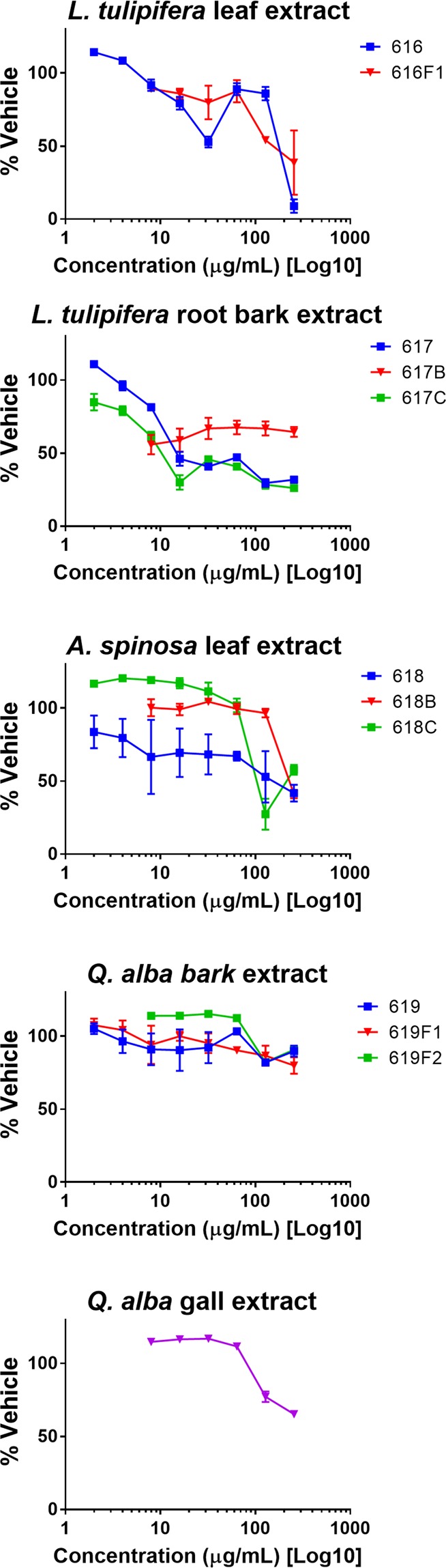
Cytotoxicity of Civil War samples exhibiting bioactivity in antibacterial models of growth, biofilm inhibition or quorum quenching. Percent keratinocyte survival is relative to vehicle control.

Of the 19 extracts studied, 13 were recognized to have potential antibiotic activity and were tested with human keratinocytes (HaCaT) to counter test for cytotoxicity. *L. tulipifera* root bark extracts 617 and 617 C displayed high levels of cytotoxicity (IC_50_ 16 μg/mL in each case). *Q. alba* extracts displayed no significant cytotoxicity at test concentrations (2–256 μg/mL). Figure 4 displays cytotoxicity across serial dilutions of samples tested; IC_50_ and IC_90_ values are reported in Supplementary Table S4.

### Chemical analysis

**Figure 5:**
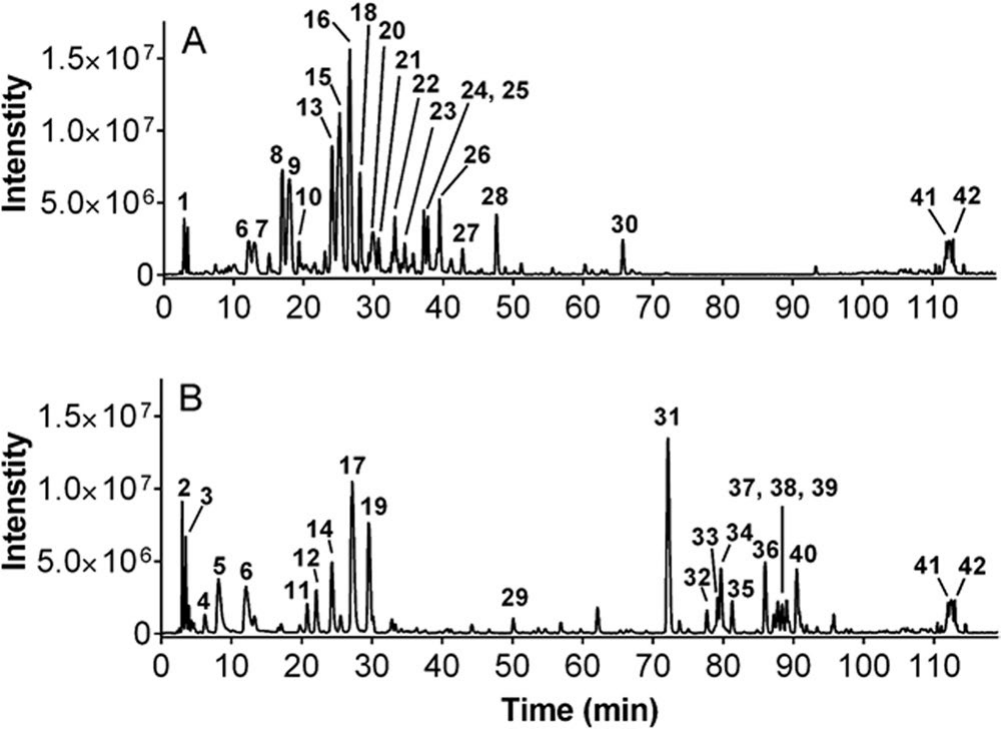
Negative ESI mass spectrum base peak chromatograms of (**A**) 619-F2 and (**B**) 620. Peaks in common are 6, 41, and 42.

*Q. alba* extracts 619-F2 and 620 were selected for chemical analysis because of their strong antibacterial activity both in growth inhibition and in adjuvant assays and because of their lack of toxicity towards human cells. Initial HPLC indicated a wealth of early eluting compounds, so the chromatographic conditions were adjusted for LC-FTMS to achieve greater separation in that region. LC-FTMS revealed that 619-F2 and 620 have few compounds in common (Fig. 5).

**Figure 6:**
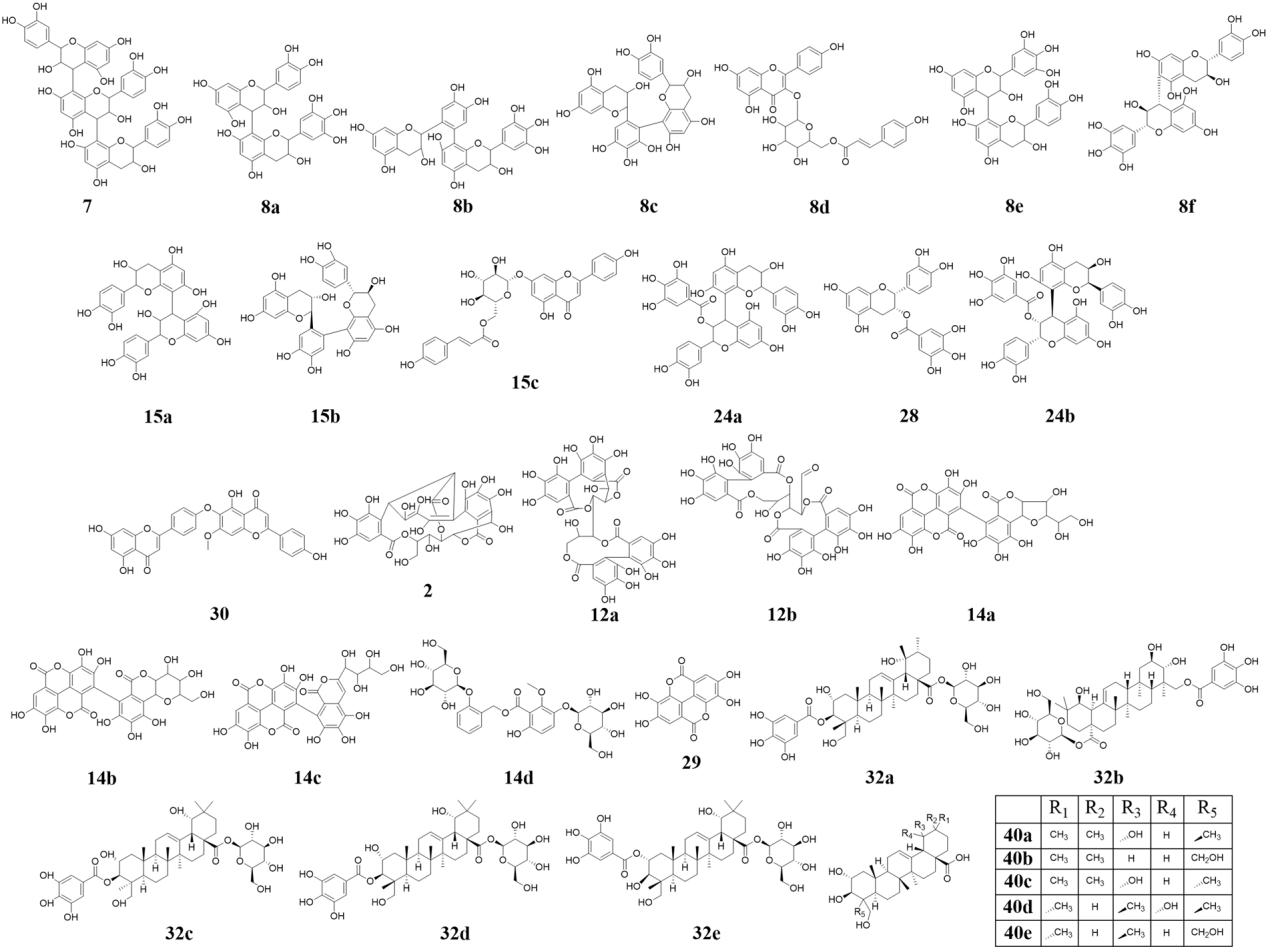
Putative compounds from fraction 619-F2 and extract 620 identified from database searches (7) isomers of procyanidin: procyanidin C_1_, procyanidin C_2_, procyanidin T_2_, procyanidin T_3,_ (**8a**) catechin-gallocatechin-4,8-dimer, (**8b**) catechin-gallocatechin-6′,8-dimer, (**8c**) gallocatechin-catechin-6′,8-dimer, (**8d**) potengriffioside A and tiliroside, (**8e**) prodelphinidin C, (**8f**) (2 R,2′R,3 S,3′S,4 R)-[2′-(3,4-dihydroxyphenyl)-3,3′,4,4′-tetrahydro-2-(3,4,5 trihydroxyphenyl)-4,6′-Bi-2H-1-benzopyran]-3,3′,5,5′,7,7′-hexol, (**15a**) isomers of procyanidin B: procyanidin B_1_, procyanidin B_2_, procyanidin B_3_, procyanidin B_4_, procyanidin B_5_, procyanidin B_6_, procyanidin B_7_, procyanidin B_8_, (**15b**) catechol-catechol-6′,8-dimer, (**15c**) echinacin, (**24a**) isomers of procyanidin B 3-O-gallate: procyanidin B_1_ 3-O-gallate, procyanidin B_2_ 3-O-gallate, procyanidin B_3_ 3-O-gallate, (**24b**) procyanidin B_2_ 3′-O-gallate, (**28**) epicatechin gallate, (**30**) isocryptomerin (**2**) castalin and vescalin, (**12a**) casuariin, (**12b**) pedunculagin, (**14a**) castacrenin A, (**14b**) castacrenin B, (**14c**) castacrenin C, (**14d**) leiocarposide, (**29**) ellagic acid, (**32a**) 2, 19, 23-trihydroxy-3-[(3, 4, 5-trihydroxybenzoyl) oxy]-β-D-glucopyranosyl ester (2α, 3β, 4α)-urs-12-en-28-oic acid and 2, 19, 23-trihydroxy-3-[(3, 4, 5-trihydroxybenzoyl) oxy]-α-D-glucopyranosyl ester (2α, 3β, 4α)-urs-12-en-28-oic acid, (**32b**) quercotriterpenoside I, (**32c**) quercotriterpenoside II, (**32d**) quercotriterpenoside III, (**32e**) quercotriterpenoside VI, (**40a**) arjugenin, (**40b**) belleric acid, (**40c**) sericic acid, (**40d**) 2α,19,23-trihydroxyursolic acid, (**40e**) 2,3,23,24-tetrahydroxy-(2α,3β)-urs-12-en-28-oic acid.

Analysis of LC-FTMS revealed 22 peaks in 619-F2 and 24 peaks in 620 with ≥1% peak area. Of these peaks, 16 and 10 respectively were putatively matched with known *Quercus* spp. Compounds (Fig. 6). Only three compounds were found in both 619-F2 and 620, 6, 41, and 42 with *m/z* of 466.0306, 367.2866, and 367.2866 respectively.

## Discussion

Extracts of *L. tulipifera*, *A. spinosa*, and *Q. alba* displayed inhibitory activity against bacteria that cause skin and soft tissue infections, substantiating their use as antiseptics during the American Civil War. These medicinal plants may be useful in modern medicine as treatments for antibiotic-resistant bacteria. Of particular interest are 618B and 620 as *S. aureus* biofilm inhibitors and 619, 619-F2, and 620 as growth inhibitors of carbapenem-resistant *Klebsiella pneumoniae*.

While a 1947 survey of antibacterial properties of plants found no activity in *A. spinosa* and *L. tulipifera*^15^, the positive results in this experiment may be explained by differences in a number of factors. The previous study used H_2_O extracts whereas this experiment used MeOH extracts^15^; *L. tulipifera* bark was historically prepared for treatment by dissolving in EtOH^5^, which produces an extraction profile similar to MeOH^16^. Additionally, given the role of endophytic microorganisms in the synthesis of secondary metabolites, the chemical composition of plant extracts can vary based on differences in the plant microbiome^17^. Other possible sources of variation include collection date and location, assay method, and extract concentration tested. Finally, given the variability in how different laboratories may perform one type of extraction, results can vary between related studies. For example, of two studies that evaluated *Aralia nudicaulis* root (a traditional Native American remedy ingredient) for growth inhibition of mycobacteria, only one reported moderate antibacterial effects while the other reported little activity^18,19^.

In his report, Porcher recommended the entire genus *Quercus* as a source of antiseptics^5^. This activity is confirmed not only by the results of the experiments reported herein, but also by multiple other studies showing antibiotic effects by *Quercus* spp. extracts^20–24^. A European herbal remedy referred to as *Quercus cortex* (originating from *Q. robur*, *Q. petrea*, and *Q. pubescens* bark) has shown weak antibacterial and quorum sensing inhibition effects^25^. Acorn extract from a variety of oaks has shown inhibition of both Gram-positive and Gram-negative bacteria^26^.

However, the activity of various *Quercus* spp. extracts is far from uniform. For example, the *Q. alba* gall extract (620) in this study inhibited growth of drug-resistant *K. pneumoniae* whereas a study of *Q. infectoria* galls found no significant inhibition of drug-resistant *K. pneumoniae*^24^.

Antibacterial activity in oak extracts is frequently attributed to tannins^27^, compounds that typically interfere with biological processes by binding to proteins^28^. In *Quercus*, tannin content is typically highest in galls, with a reported 70% tannin content in *Q. infectoria* galls^27^. In this experiment, higher activity in 620 (gall crude extract) over 619 (bark crude extract) and 619-F2 (bark tannin fraction) over 619-F1 (bark non-tannin fraction) suggests that *Q. alba*’s growth inhibitory activity is due to tannins. However, quorum sensing inhibition by 619-F1 suggests that additional compounds could contribute to the antibacterial activity of crude oak extract, the medicine used during the Civil War.

LC-FTMS analysis of 619-F2 and 620 confirmed the existence of a variety of tannins in both extracts (Supplementary Tables S6 and S7). Of particular interest are ellagitannin isomers, 2, found in 620; as well as related ellagitannins 12a and 12b. Ellagitannins have been reported to have antibiotic activity against antibiotic-resistant *S. aureus*^9^. While only three MS peaks were found in common between 619-F2 and 620, both extracts are rich in tannins. 619-F2 is enriched in procyanidin condensed tannins and 620 contains many ellagitannins and triterpenes.

Tannins have been shown to inhibit growth in a wide range of bacteria, fungi, and viruses. Suggested mechanisms of action include inactivation of microbial enzymes, inhibition of membrane transport, and sequestering essential metal ions in complexes^28^. Tannins may also act as biofilm inhibitors by binding to matrix proteins^29^. However, tannins have also been found to bind with digestive enzymes and nutrients such as proteins and starches, and as such are generally considered as anti-nutritive; a variety of animals have shown gastrointestinal distress and decreased growth when fed on high-tannin diets^28^. Because of this nondiscriminatory binding, external applications of *Q. alba* extracts would be preferable to internal or systemic applications; Porcher recommended that powdered oak bark be applied in a wash for gangrene and a poultice for wounds^5^.

Leaves of several *Quercus* species (*Q. cerris*, *Q. ilex*, *Q. virginiana*, *Q. incana*) have also shown antibacterial properties, including biofilm and quorum sensing inhibition^20,22,30^. One future research direction could be to compare the antibacterial properties of *Q. alba* leaves with the activity identified in bark and gall extracts.

While *A. spinosa* has several reported uses in traditional medicine^31^, it has not frequently been studied for medicinal properties. The most notable results of this experiment for *A. spinosa* are significant biofilm inhibition by 618B (leaf hexane partition) and quorum sensing inhibition by 618 C (leaf ethyl acetate partition). The presence of these adjuvant properties rather than simple growth inhibitory activity in *A. spinosa* leaves may explain the 1947 report of no significant antibiotic activity in *A. spinosa*^15^.

Other *Aralia* species have exhibited antibacterial activity in roots^18^ and aerial parts (flowers, leaves, and stems)^32^, including biofilm inhibition by *A. cachemirica*^32^. In his list, Porcher also ascribed antiseptic activity to *A. racemosa*^5^.

*L. tulipifera* has been widely studied and its various parts have exhibited a variety of medicinal effects including antibacterial^33^, anti-malarial^34^, and anti-cancer^35,36^ activity. The other species of *Liriodendron*, *L. chinense*, is used in Chinese traditional medicine and has been shown to have antibacterial effects^37^. Additionally, an extract from a hybrid of *L. tulipifera* and *L. chinense* has been shown to exhibit inhibition of biofilm production and quorum sensing^38^.

In the present study, *L. tulipifera* extracts demonstrated activity in the inhibition of growth, biofilm production, and quorum sensing. However, the root bark extract (617), which is generally more bioactive than the leaf extract (616) and branch bark extract (621) in our models, displayed significant mammalian cytotoxicity (IC_50_: 16 µg/mL). It may therefore be ill-suited for medicinal use, or at least dose-limited. A study of *L. tulipifera* for antiplasmodial activity also found high cytotoxicity in active fractions but it has been suggested that, given the use of *L. tulipifera* in traditional medicine, toxicity may not be problematic *in vivo* at therapeutic doses^34^. Porcher recommended root bark as the medicinal part of *L. tulipifera* to be harvested^5^; perhaps preparation techniques or dosage made the potency/toxicity trade-off worthwhile in a wartime context. Interestingly, Porcher also suggested *L. tulipifera* bark as a substitute for *Cinchona* bark in malaria treatment, an application supported by recent research^34^.

Perhaps the most notable *L. tulipifera* extract with low toxicity is 616-F1 (leaf non-tannin fraction), which displayed little growth inhibition but significant biofilm and quorum sensing inhibition—an adjuvant effect similar to the *A. spinosa* extracts tested.

Further study should focus on bioassay-guided fractionation, a recursive process of fractionation and bioassay to identify individual active compounds and synergistic relationships. Of the extracts tested, 616-F1, 618B, 618 C, 619-F2, and 620 exhibit the most promise for antibiotic NCEs and are good candidates for this process. Specifically, the HPLC methods developed for 619-F2 and 620 could be used to produce further fractions with adaptation to preparative liquid chromatography.

*In vivo* testing of the antibacterial properties of extracts active *in vitro* is also a logical next step in this research. Given the potential of some of these extracts as adjuvants rather than direct antibiotics, they may be tested as adjuvants with existing, FDA-approved antibiotics for the potentiation of antibacterial activity in wound infections.

Finally, given the activity seen in the extracts tested in this study, it may be worthwhile to investigate the antibacterial properties of other plants recorded as antiseptics in Porcher’s book. In total, 37 plant species were described as having antiseptic applications^5^. As the global spread of antibiotic-resistant strains of bacteria continues, it is increasingly important to consider all possible sources of new, and perhaps old, treatments.

## Methods

### Plant material

Samples of *Liriodendron tulipifera*, *Aralia spinosa*, and *Quercus alba* were identified and collected in May 2015 from Lullwater Preserve on the Emory University campus in Atlanta, Georgia. Leaves were gathered manually and a handsaw was used to cut segments of roots and branches for bark collection. Vouchers (Accession numbers 20338-20341) were deposited in the Emory University Herbarium (GEO) in Atlanta and digital copies of the specimens are accessible for viewing online via the SERNEC web portal^39^. Samples were dried and ground into powder by either a Wiley mill equipped with a 2 mm mesh or coffee grinder (Table 1).

### Extraction, partitioning, and fractionation

All ground material (Table 1) was sonicated in MeOH (1 g/10 mL). After 20 minutes the sample was filtered sequentially with Whatman filter paper 8 and 2, and then fresh MeOH was added to the plant material for a second round of sonication. The two filtrates were combined and dried *in vacuo* at ≤40 °C. The resulting residue was suspended in H_2_O, frozen, and lyophilized. The dried extract was collected and 20 mg of each extract was dissolved in DMSO (10 mg/mL) for biological testing.

Extracts 617 and 618 were suspended in H_2_O (1 g/10 mL) and were sequentially partitioned in hexane, ethyl acetate, and *n*-butanol, yielding 4 partitions. Extracts 616 and 619 were dissolved in 95% ethanol (1 g/2 mL and 1 g/3 mL, respectively), loaded on a Sephadex LH-20 column (25 g, 32 × 2.5 cm), and sequentially eluted with 95% ethanol (300 mL), 70% acetone (300 mL), and 100% acetone (150 mL) to yield three fractions. All partitions and fractions were dried *in vacuo*, resuspended in H_2_O, frozen and lyophilized before being dissolved in DMSO (10 mg/mL) for biological testing.

### Bacterial strains and growth conditions

In this study, six strains of *Staphylococcus aureus* (UAMS1, UAMS929, NRS385, AH1747, AH1677, AH430, AH1872), one strain of *Staphylococcus epidermidis* (NRS101), three strains of *Klebsiella pneumoniae* (NR-15410, NR-15411, NR-15412), eight strains of *Acinetobacter baumannii* (AB5075, NR-17786, AR-BANK#0035, AR-BANK#0037, AR-BANK#0045, AR-BANK#0300, OIFC143, H72721), and one strain of *Pseudomonas aeruginosa* (AH071) were used (Supplementary Table S5). To create liquid cultures for all assays, strains were grown overnight in tryptic soy broth (TSB) with constant shaking (230 rpm). All strains were maintained on Tryptic Soy Agar (TSA) and tested in Cation-Adjusted Mueller-Hinton Broth (CAMHB).

### Growth inhibition assays

Assays were carried out under CLSI M100-S23 guidelines^40^. A working culture was created by standardizing liquid culture using a BioTek Cytation3 and inoculating into CAMHB to a concentration of 5.0 × 10^5^ CFU/mL. Working culture was added to extracts and controls in 96-well microtiter plates (Grenier-Bio 655-185) such that each well contained a total volume of 0.2 mL. Vehicle controls and antibiotic controls (ampicillin, kanamycin, and vancomycin for *Staphylococcus* spp. assays, gentamicin, tetracycline, and meropenem for other species, 0.5 to 64 µg/mL) were included for each strain. Extracts and vehicle were tested at a concentration range of 2.0 to 256 µg/mL, using 2-fold serial dilution. Plates were incubated at 37 °C, with *S. aureus, S. epidermidis*, and *P. aeruginosa* for 18 hours and *A. baumannii* and *K. pneumoniae* for 22 hours. Optical density (OD_600_) was measured using a BioTek Cytation3 plate reader at initial and final time points, to account for extract colour. The IC_50_ for growth was defined as the lowest concentration at which an extract displayed ≥50% inhibition and MIC (IC_90_) at ≥90% inhibition.

Extracts active against multidrug-resistant *A. baumannii* (OIFC143) and *K. pneumoniae* (NR-15410) were tested for growth inhibition of *S. epidermidis* and additional strains of *A. baumannii* and *K. pneumoniae*.

### Biofilm inhibition assays for *S. aureus*

Biofilm inhibition of *S. aureus* was performed as described previously^41^. Briefly, supplemented TSB with 3% NaCl, 0.5% dextrose, and 2% human plasma was used in 96-well microtiter plates (Falcon 35–1172). Working cultures of UAMS-1 (*wt*) and UAMS-929 (isogenic *ΔsarA* mutant of UAMS-1) were standardized to a concentration of 5 × 10^5^ CFU/mL and the final well volume was 0.2 mL. Extracts were assessed at sub-IC_50_ concentrations for growth, ranging from 2.0 to 256 µg/mL. The vehicle and positive control, 220D-F2, were assessed from 2.0 to 256 µg/mL. All experiments were incubated statically at 37 °C for 22 hours. Optical density (OD_600_) was measured using a BioTek Cytation3 plate reader at initial and final time points, to account for extract colour. Biofilms were rinsed twice with 1X PBS, fixed with 100% EtOH, and stained with crystal violet. The dry stain was eluted with ethanol, diluted in PBS, and quantified at 595 nm using a BioTek Cytation 3 plate reader. The MBIC_50_ (minimum biofilm inhibitory concentration) was defined as the lowest concentration at which an extract displayed ≥50% inhibition and MBIC_90_ at ≥90% inhibition.

### Quorum quenching assays for *S. aureus*

Examination of the quorum quenching potential of extracts against *S. aureus* was conducted as previously described^3^. Briefly, all *agr* fluorescent reporter strains were maintained in chloramphenicol (10 µg/mL) supplemented TSA and TSB. The assay was conducted in tissue culture-treated clear bottom, black-sided 96-well microtiter plates (Costar 3603) with a final well volume of 0.2 mL. Extracts were assessed at sub-MIC_50_ concentrations, ranging from 0.5 to 64 µg/mL. Vehicle and positive control, 224C-F2, were also assessed from 0.5 to 64 µg/mL. Plates were incubated at 37 °C in a humidified chamber, shaking at 1200 rpm (Stuart SI505). OD (600 nm) and fluorescence (493 nm excitation, 535 nm emission) readings were taken at initial (0 hr) and final (22 hr) time points.

### Cytotoxicity assays

Human immortalized keratinocytes (HaCaT) were maintained and used to examine the cytotoxicity of the active extracts with an LDH cytotoxicity assay (G-Biosciences, St. Louis, MO) as previously described^3^. Briefly, the cell culture was standardized to 4 × 10^4^ cells/mL using a hemocytometer and 0.2 mL added per well in a tissue culture treated 96-well microtiter plate (Falcon 35–3075). Plates were incubated for 48 hours to allow for seeding, and then cells were exposed to fresh media with treatment. Extracts and vehicle were serially diluted 2-fold (2–256 μg/mL) and were processed 24 hours post-treatment following manufacturer’s protocol for chemical induced cytotoxicity.

### Chemical analysis

HPLC methods were adapted from Mämmelä^42^ and were performed on an Agilent 1260 Infinity system running OpenLab CDS ChemStation (Agilent Technologies, Santa Clara, CA, USA) with an Agilent Zorbax Eclipse XDB-C18 (250 × 4.6 mm, 5 µm) column with compatible guard column at 35 °C. A gradient elution consisting of mobile phases (A) 1% formic acid in H_2_O and (B) 1.0% formic acid in MeOH at 1.0 mL/min beginning at 95:5 A:B for 9 min, then following a linear gradient to 0:100 A:B at 69 min, which was held for 9 min, before returning to initial conditions to equilibrate the column. Extracts were prepared for HPLC at 10 mg/mL in DI H_2_O with an injection volume of 10 µL.

The liquid chromatography-Fourier transform mass spectrometry (LC-FTMS) analysis was performed using a Shimadzu SIL-ACHT and Dionex 3600 SD HPLC pump with a modification of the previous method. A 10 µL injection at ambient temperature with (A) 1.0% formic acid in H2O and (B) 1% formic acid in MeOH at a flow rate of 1.0 mL/min. Initial conditions were 95:5 (A:B) and held for 9 min, changing to 38:62 (A:B) using a linear gradient at 85 min, and then 100% B at 109 min, which was held for 10 min before returning to initial conditions to equilibrate the column. The data was acquired in MS^1^ mode scanning from a *m/z* of 150–1500 on a Thermo Scientific LTQ-FT Ultra MS in negative ESI mode and processed with Thermo Scientific Xcalibur 2.2 software (San Jose, CA). The capillary temperature was 275.0 °C, nitrogen was the sheath gas at a flow of 60, source voltage and current 5.0 kV and 100.0 µA, and the capillary voltage −19.0 V.

Using SciFinder Scholar (Chemical Abstracts Service, Columbus, OH, USA) all reported compounds from the genus *Quercus* were searched for matches to the LC-FTMS accurate mass data for each peak. The resulting putative compounds for samples 619-F2 and 620 are listed in Supplementary Tables S6, S7.

## Supplementary information

Supplementary Data

## Data Availability

All data generated or analysed during this study are included in this published article and its Supplementary Information Files.
