# Supplementary material for: American Civil War plant medicines inhibit growth, biofilm formation, and quorum sensing by multidrug-resistant bacteria
Source: Sci Rep. 2019 May 22;9:7692. doi: 10.1038/s41598-019-44242-y (PMC6531439; doi:10.1038/s41598-019-44242-y)
Supplement: Supplementary file 1 — Supplementary Data [file 41598_2019_44242_MOESM1_ESM.docx]

**American Civil War plant medicines inhibit growth, biofilm formation, and quorum sensing by multidrug-resistant bacteria**

Micah Dettweiler, James Lyles, Kate Nelson, Brandon Dale, Ryan Reddinger, Daniel Zurawski, Cassandra L. Quave

**Supplementary Information**

**Supplementary Table S1.** Percent yield of partitions and fractions from dry plant mass.

| Extract | Description | Percent Yield |
| --- | --- | --- |
| 616F1 | *L. tulipifera* leaf non-tannin | 1.81 |
| 616F2 | *L. tulipifera* leaf tannin | 0.15 |
| 617B | *L. tulipifera* root bark hexane | 2.35 |
| 617C | *L. tulipifera* root bark ethyl acetate | 0.82 |
| 617D | *L. tulipifera* root bark *n*-butanol | 2.58 |
| 617E | *L. tulipifera* root bark water | 2.36 |
| 618B | *A. spinosa* leaf hexane | 0.60 |
| 618C | *A. spinosa* leaf ethyl acetate | 2.69 |
| 618D | *A. spinosa* leaf *n*-butanol | 3.90 |
| 618E | *A. spinosa* leaf water | 3.10 |
| 619F1 | *Q. alba* bark non-tannin | 4.09 |
| 619F2 | *Q. alba* bark tannin | 3.43 |

**Supplementary Table S2.** Inhibition of UAMS1 *S. aureus* biofilm formation by Civil War samples. MBIC values expressed as concentrations (μg/mL) that inhibit 50% and 90% of biofilm.

| Extract | MBIC_50_ | MBIC_90_ |
| --- | --- | --- |
| 616 | 256 | - |
| 616F1 | 32 | - |
| 616F2 | 128 | 256 |
| 617 | 2 | - |
| 617B | 8 | - |
| 617C | - | - |
| 617D | - | - |
| 617E | - | - |
| 618 | - | - |
| 618B | 2 | 32 |
| 618C | - | - |
| 618D | 256 | 256 |
| 618E | - | - |
| 619 | - | - |
| 619F1 | 256 | - |
| 619F2 | 1 | 8 |
| 619W | 32 | 32 |
| 620 | 4 | 16 |
| 620W | 16 | - |
| 621 | 64 | 256 |
| 220D-F2 | 8 | 16 |

**Supplementary Table S3.** Inhibition of *S. aureus* quorum sensing by Civil War samples. Reported here are IC_50_ values, concentrations at which 50% of fluorescence is inhibited relative to the vehicle control.

| Sample | AH430  *agr* II | AH1677  *agr* I | AH1747  *agr* III | AH1872  *agr* IV |
| --- | --- | --- | --- | --- |
| 616 | - | - | - | - |
| 616F1 | - | - | 32 | - |
| 616F2 | - | - | - | - |
| 617 | - | - | - | - |
| 617B | - | - | - | - |
| 617C | - | 32 | 16 | - |
| 617D | - | - | - | - |
| 617E | - | - | - | - |
| 618 | - | - | - | - |
| 618B | - | - | - | - |
| 618C | 8 | - | 32 | - |
| 618D | - | - | - | - |
| 618E | - | - | - | - |
| 619 | - | - | - | - |
| 619F1 | - | - | 16 | - |
| 619F2 | 64 | - | - | - |
| 619W | - | - | - | - |
| 620 | - | - | - | - |
| 621 | - | - | 32 | - |
| 224CF2 | 2 | 64 | 8 | 64 |

**Supplementary Table S4.** Growth inhibition of human keratinocytes (HaCaTs) by *L. tulipifera*, *A. spinosa*, and *Q. alba* samples. IC_50_ is the minimum concentration required to inhibit 50% of growth and was calculated relative to the vehicle control. A maximum concentration of 256 µg/mL was tested.

|  | IC_50_ | IC_90_ |
| --- | --- | --- |
| 616 | 256 | 256 |
| 616F1 | 256 | >256 |
| 617 | 16 | >256 |
| 617B | >256 | >256 |
| 617C | 16 | >256 |
| 618 | 256 | >256 |
| 618B | 256 | >256 |
| 618C | 128 | >256 |
| 619 | >256 | >256 |
| 619F1 | >256 | >256 |
| 619F2 | >256 | >256 |
| 620 | >256 | >256 |
| 621 | >256 | >256 |

**Supplementary Table S5.** Bacterial strains utilized.

| Strain | Species | Strain ID | Characteristics |
| --- | --- | --- | --- |
| UAMS1 | *Staphylococcus aureus* | ATCC49230 | MRSA isolate from human osteomyelitis |
| UAMS929 | *Staphylococcus aureus* |  | Biofilm-deficient mutant |
| NRS385 | *Staphylococcus aureus* | USA500 | MRSA, also resistant to ERY, CLIN, TET, SXT, LEV, GM |
| AH430 | *Staphylococcus aureus* | SA502a + pDB59 cmR | *agr* type II YFP reporter |
| AH1677 | *Staphylococcus aureus* | AH845 + pDB59 cmR | *agr* type I YFP reporter |
| AH1747 | *Staphylococcus aureus* | MW2 + pDB59 cmR | *agr* type III YFP reporter |
| AH1872 | *Staphylococcus aureus* | MN EV(AH407) + pDB59 cmR | *agr* type IV YFP reporter |
| NRS101 | *Staphylococcus epidermidis* | ATCC35984 | biofilm producer |
| AH71 | *Pseudomonas aeruginosa* | PAO1 |  |
| EU24 | *Acinetobacter baumannii* | NR-17786 | isolate from blood |
| EU27 | *Acinetobacter baumannii* | OIFC143 | isolate from human thigh wound |
| EU35 | *Acinetobacter baumannii* | H72721 | isolate from human sputum |
| AB5075 | *Acinetobacter baumannii* | AB5075 | laboratory strain |
| CDC0035 | *Acinetobacter baumannii* | AR-BANK#0035 | carbapenem resistant |
| CDC0037 | *Acinetobacter baumannii* | AR-BANK#0037 | carbapenem resistant |
| CDC0045 | *Acinetobacter baumannii* | AR-BANK#0045 | carbapenem resistant |
| CDC0300 | *Acinetobacter baumannii* | AR-BANK#0300 |  |
| EU32 | *Klebsiella pneumoniae* | NR-15410 | carbapenem resistance from *bla*_KPC_ gene |
| EU33 | *Klebsiella pneumoniae* | NR-15411 | carbapenem resistance from *bla*_KPC_ gene |
| EU34 | *Klebsiella pneumoniae* | NR-15412 | carbapenem resistance from *bla*_KPC_ gene |

**Supplementary Table S6.** Putative matches for compounds identified by negative ESI FT-MS from fraction 619-F2. Peak number corresponds with peak numbers in Figure 5. The parent ion used for MS^2^ and putative structure masses is indicated in **bold**.

| Peak No. | Retention Time (min) | Relative Abundance (%) | *m/z* | MS^2^ | Formula (Δppm) | Putative Matches |
| --- | --- | --- | --- | --- | --- | --- |
| 1 | 2.89 | 1.2 | **383.1211,** 459.1375, 533.1756, 549.1702 | 191.1512 | C_14_H_23_O_12_ (3.7) | no matches |
| 6 | 12.13 | 2.3 | **466.0306,** 933.0721 | 301.0781, 457.1463 | C_8_H_18_O_22_ (2.4) | no matches |
| 7 | 12.95 | 2.4 | 865.2027 | 575.2056, 695.2256, 713.1812 | C_45_H_37_O_18_ (4.8) | procyanidin C_1_, procyanidin C_2_, procyanidin T_2_, procyanidin T_3_ |
| 8 | 16.98 | 5.5 | 593.1333 | 289.1368, 407.1845, 425.1403 | C_30_H_25_O_13_ (5.6) | catechin-gallocatechin-4,8-dimer, catechin-gallocatechin-6',8-dimer, gallocatechin-catechin-6',8-dimer, potengriffioside A, prodelphinidin C, tiliroside, (2R,2'R,3S,3'S,4R)-[2'-(3,4-dihydroxyphenyl)-3,3',4,4'-tetrahydro-2-(3,4,5 trihydroxyphenyl)-4,6'-Bi-2H-1-benzopyran]-3,3',5,5',7,7'-hexol |
| 9 | 17.89 | 7.6 | 593.1332 | 289.1397, 407.1963, 425.1470, 467.1748 | C_30_H_25_O_13_ (5.4) | see peak 9 |
| 10 | 19.35 | 1.1 | 593.1322 | 305.1477, 423.1770, 441.1596, 467.2286, 575.2450 | C_30_H_25_O_13_ (3.7) | see peak 9 |
| 13 | 24.02 | 6.9 | **602.0666,** 1205.1498 | 457.2881, 915.0694 | C_12_H_26_O_27_ (0.0) | no matches |
| 15 | 25.17 | 12.6 | 577.1385 | 289.1338, 407.1862, 425.1578, 451.1998 | C_30_H_25_O_12_ (5.6) | procyanidin B1, procyanidin B2, procyanidin B3, procyanidin B4, procyanidin B5, procyanidin B6, procyanidin B7, procyanidin B8, catechol-catechol-6',8-dimer, echinacin |
| 16 | 26.61 | 11.8 | 577.1384 | 289.1413, 407.1700, 425.1431, 451.1729, 559.2598 | C_30_H_25_O_12_ (4.3) | see peak 16 |
| 18 | 28.04 | 4.4 | 865.206 | 577.2230, 695.2471, 739.1813 | C_45_H_37_O_18_ (8.6) | see peak 7 |
| 20 | 29.95 | 3.1 | 577.1381 | 289.1374, 407.1706, 425.1357, 451.1759 | C_30_H_25_O_12_ (5.0) | see peak 16 |
| 21 | 30.72 | 1.3 | 602.0654 | 457.2701 | C_37_H_14_O_9_ (1.8) | no matches |
| 22 | 33.06 | 2.1 | 577.138 | 407.1593, 425.1190, 451.1545 | C_30_H_25_O_12_ (5.0) | see peak 16 |
| 23 | 34.48 | 1.2 | 577.1374 | 407.1684, 425.1382, 451.1536 | C_30_H_25_O_12_ (3.9) | see peak 16 |
| 24 | 37.21 | 2.3 | 729.1506 | 407.1790, 425.1365, 559.1947, 577.1635 | C_37_H_29_O_16_ (6.1) | procyanidin B2 3'-O-gallate, procyanidin B1 3-O-gallate, procyanidin B3 3-O-gallate, procyanidin B2 3-O-gallate |
| 25 | 37.79 | 2.2 | **729.1510**, 775.1578 | 407.1631, 451.1956, 559.0828, 577.1213, 603.1504 | C_37_H_29_O_16_ (6.3) | see peak 27 |
| 26 | 39.47 | 3.7 | **577.1383**, 623.1454, **865.2057** | 289.1422, 407.1887, 425.1331, 451.2100, 559.2145 | C_30_H_25_O_12_ (5.5) | see peak 16 |
| 27 | 42.75 | 1.1 | 617.0522 | 450.3011, 573.4445, 595.3807, | C_37_H_13_O_10_ (1.3) | no matches |
| 28 | 47.61 | 2.5 | **441.0848**, 487.0911, **883.1789** | 169.0292, 289.1651, 331.1760 | C_22_H_17_O_10_ (4.6) | epicatechin gallate |
| 30 | 65.67 | 1.5 | 551.1061 | 343.1511 | C_31_H_19_O_10_ (14.1) | isocryptomerin |
| 41 | 112.46 | 2.2 | **367.2866,** 599.5338 | 323.3504, 349.3503 | C_22_H_39_O_4_ (3.4) | no matches |
| 42 | 112.98 | 1 | **367.2868,** 599.5338 | 323.3834, 349.3474 | C_22_H_39_O_4_ (3.7) | no matches |

**Supplementary Table S7.** Putative matches for compounds identified by mass spectrometry from extract 620. Peak number corresponds with peak numbers in Figure 5. The parent ion used for MS^2^ and putative structure masses is indicated in **bold**.

| Peak No. | Retention Time (min) | Relative Abundance (%) | *m/z* | MS^2^ | Formula (Δppm) | Putative Matches |
| --- | --- | --- | --- | --- | --- | --- |
| 2 | 3 | 3.0 | 631.0618 | 569.2032, 613.1992 | C_27_H_19_O_18_ (6.7) | castalin, vescalin |
| 3 | 3.49 | 2.6 | 631.0617 | 569.1972, 587.1975, 613.2015 | C_27_H_19_O_18_ (6.5) | see peak 2 |
| 4 | 6.19 | 1.0 | **532.0514**, 1065.1131 | 487.2613 | C_16_H_20_O_20_ (-5.3) | no matches |
| 5 | 8.17 | 4.9 | **466.0301,** 933.07292, 979.0782 | 457.3207 | C_26_H_10_O_9_ (-3.9) | no matches |
| 6 | 12.09 | 4.9 | **466.0306,** 933.07212, 979.0788 | 457.1415 | C_26_H_10_O_9_ (-2.8) | no matches |
| 11 | 20.8 | 1.4 | 783.0716 | 301.0530, 481.2079 | C_34_H_23_O_22_ (3.7) | casuariin, pedunculagin |
| 12 | 22.06 | 2.3 | **631.0600,** 763.1038 | 493.1571, 613.1783 | C_27_H_19_O_18_ (3.6) | see peak 2 |
| 14 | 24.3 | 4.4 | 613.0497 | 523.1821, 595.1963 | C_27_H_17_O_17_ (4.1) | castacrenin A, castacrenin B, castacrenin C, leiocarposide |
| 17 | 27.19 | 11.6 | 613.0513 | 493.1871 | C_27_H_17_O_17_ (6.4)) | see peak 15 |
| 19 | 29.57 | 7.9 | 613.0507 | 493.2256, 523.1789 | C_27_H_18_O_17_ (5.6) | see peak 15 |
| 29 | 62.11 | 1.4 | **300.9997**, 603.0075, 905.0183 | no data | C_14_H_5_O_8_ (2.2) | ellagic acid |
| 31 | 72.16 | 12.9 | 711.4018 | 503.4474, 665.1741 | C_30_H_63_O_18_ (0.1) | no matches |
| 32 | 77.67 | 1.2 | 817.4049 | 313.0863 | C_43_H_62_O_15_ (3.959) | 2,​19,​23-​trihydroxy-​3-​[(3,​4,​5-​trihydroxybenzoyl)​oxy]​-​β-​D-​glucopyranosyl ester (2α,​3β,​4α)​-urs-​12-​en-​28-​oic acid and 2,​19,​23-​trihydroxy-​3-​[(3,​4,​5-​trihydroxybenzoyl)​oxy]​-​α-​D-​glucopyranosyl ester (2α,​3β,​4α)​-urs-​12-​en-​28-​oic acid, quercotriterpenoside I, quercotriterpenoside II, quercotriterpenoside III, quercotriterpenoside VI |
| 33 | 79.18 | 1.6 | **563.3250,** 709.3840 | 501.4094, 663.1527 | C_31_H_47_O_9_ (4.4) | no matches |
| 34 | 79.68 | 3.6 | 711.4001 | 503.4423, 665.1958 | C_30_H_63_O_18_ (-2.6) | no matches |
| 35 | 81.25 | 1.5 | 711.3989 | 503.4772, 665.1845 | C_30_H_63_O_18_ (-4.3) | no matches |
| 36 | 85.94 | 3.8 | 695.4056 | 487.4722, 649.1960 | C_30_H_63_O_17_ (-2.1) | no matches |
| 37 | 87.73 | 1.5 | 693.3886 | 485.4439, 647.1794 | C_37_H_57_O_12_ (4.3) | no matches |
| 38 | 88.39 | 1.5 | 695.4041 | 487.4730, 649.1933 | C_37_H_59_O_12_ (4.2) | no matches |
| 39 | 89.04 | 1.8 | 693.3888 | 485.4452, 647.1807 | C_30_H_61_O_17_ (-3.8) | no matches |
| 40 | 90.45 | 4.4 | **503.3404**, 549.3470, 1007.6940 | 529.1687 | C_30_H_47_O_6_ (5.1) | arjugenin, belleric acid, sericic acid, 2α,19,23-trihydroxyursolic acid, 2,3,23,24-tetrahydroxy-(2α,3β)-urs-12-en-28-oic acid |
| 41 | 112.45 | 2.6 | 367.2866 | 323.3701, 349.3576 | C_22_H_39_O_4_ (3.3) | no matches |
| 42 | 112.95 | 1.0 | 367.2866 | 323.3366 | C_22_H_39_O_4_ (3.3) | no matches |
| 43 | 120.01 | 2.1 | **571.5004,** 581.5298 | 551.1280, 553.1331 | C_37_H_65_O_3_N (6.0) | no matches |
